# Novel marine actinobacteria from emerald Andaman & Nicobar Islands: a prospective source for industrial and pharmaceutical byproducts

**DOI:** 10.1186/1471-2180-13-145

**Published:** 2013-06-22

**Authors:** Balakrishnan Meena, Lawrance Anbu Rajan, Nambali Valsalan Vinithkumar, Ramalingam Kirubagaran

**Affiliations:** 1 Andaman and Nicobar Centre for Ocean Science and Technology, ESSO-NIOT, Dollygunj, Port Blair, Andaman and Nicobar Islands 744 103, India; 2 Marine Biotechnology Group, ESSO-National Institute of Ocean Technology (NIOT), Ministry of Earth Sciences (Govt. of India), Chennai 600 100, India

**Keywords:** Andaman & Nicobar Islands, Marine actinobacteria, Enzymatic activity, Hemolytic activity, Antibacterial activity

## Abstract

**Background:**

Andaman and Nicobar Islands situated in the eastern part of Bay of Bengal are one of the distinguished biodiversity hotspot. Even though number of studies carried out on the marine flora and fauna, the studies on actinobacteria from Andaman and Nicobar Islands are meager. The aim of the present study was to screen the actinobacteria for their characterization and identify the potential sources for industrial and pharmaceutical byproducts.

**Results:**

A total of 26 actinobacterial strains were isolated from the marine sediments collected from various sites of Port Blair Bay where no collection has been characterized previously. Isolates were categorized under the genera: *Saccharopolyspora*, *Streptomyces, Nocardiopsis*, *Streptoverticillium*, *Microtetraspora*, *Actinopolyspora*, *Actinokineospora* and *Dactylosporangium*. Majority of the isolates were found to produce industrially important enzymes such as amylase, protease, gelatinase, lipase, DNase, cellulase, urease and phosphatase, and also exhibited substantial antibacterial activity against human pathogens. 77% of isolates exhibited significant hemolytic activity. Among 26 isolates, three strains (NIOT-VKKMA02, NIOT-VKKMA22 and NIOT-VKKMA26) were found to generate appreciable extent of surfactant, amylase, cellulase and protease enzyme. NIOT-VKKMA02 produced surfactant using kerosene as carbon source and emulsified upto *E*_24_–63.6%. Moreover, NIOT-VKKMA02, NIOT-VKKMA22 and NIOT-VKKMA26 synthesized 13.27 U/ml, 9.85 U/ml and 8.03 U/ml amylase; 7.75 U/ml, 5.01 U/ml and 2.08 U/ml of cellulase and 11.34 U/ml, 6.89 U/ml and 3.51 U/ml of protease enzyme, respectively.

**Conclusions:**

High diversity of marine actinobacteria was isolated and characterized in this work including undescribed species and species not previously reported from emerald Andaman and Nicobar Islands, including *Streptomyces griseus*, *Streptomyces venezuelae* and *Saccharopolyspora salina*. The enhanced salt, pH and temperature tolerance of the actinobacterial isolates along with their capacity to secrete commercially valuable primary and secondary metabolites emerges as an attractive feature of these organisms. These results are reported for the first time from these emerald Islands and expand the scope to functionally characterize novel marine actinobacteria and their metabolites for the potential novel molecules of commercial interest.

## Background

Actinobacteria, are filamentous Gram positive prokaryotes with 67-78% G + C content [1]. Actinobacteria are considered as an intermediate group of bacteria and fungi and are recognized as prokaryotic organisms. Actinobacteria are present in various ecological habitats and marine environments [2] and to cope with the environmental stress, marine microorganisms have developed a complex stress management for their survival, which is being unrevealed for multiple purposes [3]. They are being exploited for various commercial applications in environmental, biomedical and industrial sectors [4]. Various metabolites of actinobacterial origin have been reported for their excellent bioactivity [5]. Marine environment is the prime reservoir of biological diversity and the marine microorganisms are recognized to be rich sources of novel compounds. In India, about 1000 natural products were derived from marine microbes [6], in which, marine actinobacteria have been proven as a potential source of bioactive compounds and richest source of secondary metabolites. They are the most economically and biotechnologically valuable prokaryotes.

Currently, enzymes and drugs from microbial origin are substituting the chemical catalysts in leather, food, paper, pharmaceuticals and textile industries [7]. Majority of the enzymes are derived from plants, animals and microorganisms. Among them, microbes are the topmost due to their rapid doubling time and enzyme production when compared with plants or animals to meet the existing market demand for industrial enzymes [8]. Marine actinobacteria are capable of producing enzymes with good stability at higher temperature and alkaline conditions. Even though, the production of antibiotics as major bioactive compounds from marine actinobacteria [4,9] the ability to synthesize variety of industrial enzymes can be an attractive phenomenon to accomplish our future demand.

A little is known about the diversity of actinobacteria in marine sediments, which is an inexhaustible resource that has not been properly exploited. Many reports suggested that marine sediment is a rich source of actinobacteria [10]. Andaman coast in India is holding outsized diverse and unexploited ecosystem for the isolation of novel actinobacteria with effective bioactive molecules [11]. The Andaman and Nicobar (A & N) Islands marine ecosystem are mostly unexplored, and may provide a rich source of microorganisms producing novel and efficient antimicrobial compounds [12]. Only limited research on marine actinobacteria from A & N Islands has been reported. To our knowledge, no studies have been reported on the characterization of marine actinobacteria from Port Blair Bay of A & N Islands. Rather, these Islands are an unexploited part of Indian seas and have rarely been explored for microbial diversity research and their metabolites. Hence, there is an immense possibility to identify and functionally characterize new marine actinobacteria to identify novel bioactive compounds. Accordingly, the present study at Port Blair Bay of A & N Islands aimed to isolate and functionally characterize the marine actinobacteria of industrial and pharmaceutical interest with the ultimate objective of discovering novel bioactive compounds.

## Methods

### Study area

**Figure 1:**
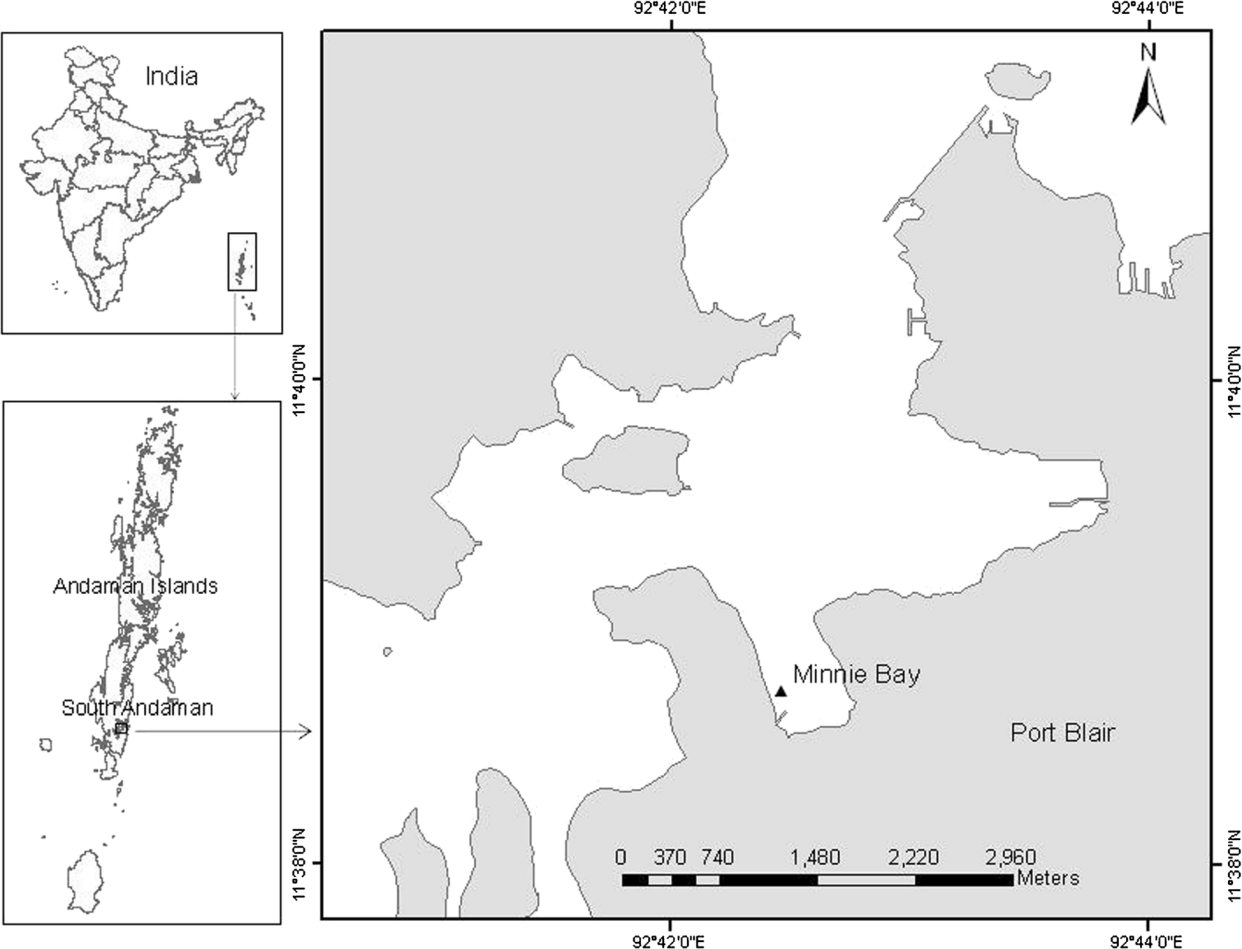
Map showing the study area, Minnie Bay, A & N Islands, India.

A & N group of Islands is consisting of 572 islands from Landfall Island to Great Nicobar stretches through the distance of 770 km length and covers an area of about 8249 Km^2^. The study area Minnie Bay, Port Blair, South Andaman, is situated at the proximal end of the Port Blair Bay (Figure 1). Two major species of mangrove *Rhizophora* sp. and *Avecenia* sp. were making most of the boundary of the bay. The study area is affected by the tidal amplitude of 1.5 to 2.0 m approximately. This Bay is found to be rich in nutrients due to the domestic waste discharges from the residential complex and degradation of submerged mangrove vegetation after the tsunami incident in 2004.

### Collection of sediment samples

Marine sediment samples were collected from Minnie Bay using Global Positioning System (GARMIN eTrex Vista H, Taiwan) coordinates of 11°38“42.8”N lat. and 92°42“30.7”E long. Samples were collected randomly in sterile polythene bags and transported immediately to the laboratory for isolation of marine actinobacteria. Based on the colony morphology, 26 distinct colonies were selected for characterization studies.

### Measurement of physico-chemical parameters

The pH of sediment samples was measured as described previously by Ramesh and Mathivanan, [13]. Briefly, 10 g of each marine sediment samples were suspended in 20 ml of distilled water and was allowed to stand for 20 min to attain the equilibrium condition. Subsequently, the pH was recorded using digital meter (Thermo Orion 420 A plus, USA) and salinity of the samples was documented with a refractometer (ATAGO S/Milli-E, USA). Temperature, Dissolved Oxygen (DO) and nutrients of the sampling site were documented as described by Grasshoff et al. [14].

### Isolation of marine actinobacteria

Isolation and enumeration of actinobacteria was performed as described previously by Ellaiah et al. [15] using starch casein agar (SCA) medium containing soluble starch 10 g, vitamin free casein 0.3 g, KNO_3_ 2 g, NaCl 2 g, K_2_HPO_4_ 2 g, MgSO_4_.7H_2_O 0.05 g, CaCO_3_ 0.02 g, FeSO_4_.7H_2_O 0.01 g and agar 20 g, pH 7.0 ± 0.2 [16], with 50% aged sea water. Medium was added with nalidixic acid 25 μg/ml (Hi Media, Mumbai, India) to inhibit the fast growing Gram negative bacteria. Soil samples were mixed and then serially diluted in sterile sea water and spread plated on SCA plates. The plates were incubated at room temperature (28 ± 2°C) for 21 days.

Appearance and growth of marine actinobacteria were monitored regularly. The colonies were recognized by their characteristic chalky to leathery appearance on SCA plates. Colonies were purified using SCA and International Streptomyces Project medium 2 (ISP2 medium) and sub cultured in SCA slants for further studies. Pure cultures were also preserved in 20% glycerol vials and stored at −80°C for long term preservation [17].

### Growth characteristics of marine actinobacteria

Actinobacterial isolates were streaked on SCA plates, incubated at room temperature, and the growth rate was monitored daily up to 21 days. Isolates which exhibited good growth within 4 days were considered as fast growers, the isolates those revealed good growth between 4^th^ to 7^th^ day of incubation were considered as moderate growers and the isolates those raised after 7 days was considered as slow growers [13]. Mycelial colour was also monitored and documented along with the growth parameters.

### Characterization and identification of actinobacteria

Morphological, biochemical, culture and physiological characterization of the actinobacterial isolates of Minnie Bay were performed as recommended by the International Streptomyces Project (ISP) which were described by Shirling and Gottileb [18]. Microscopic study was performed with cover slip culture and cellophane method [19]. Formation of aerial, substrate mycelium and spore arrangements on mycelium were monitored under a phase contrast microscope (Nikon ECLIPSE E600, USA) at 100× magnification. Culture characteristics such as growth, coloration of aerial and substrate mycelia, formation of soluble pigment were investigated in eight different media including SCA, nutrient agar, yeast malt agar (ISP-2), oat meal agar (ISP-3), inorganic salt agar (ISP-4), glycerol-asparagine agar (ISP-5), peptone yeast extract agar (ISP-6) and tyrosine agar (ISP-7) with the procedures as recommended by ISP. Biochemical characterization, namely, Gram’s reaction, MR-VP, H_2_S production, nitrate reduction, oxidase, catalase, urease, starch, casein and gelatin hydrolysis, blood hemolysis, TSI, citrate utilization, esculin and hippurate hydrolysis was also performed as suggested by ISP. Physiological characterization such as, effect of pH (5–11), growth range in NaCl (5-30%) and survival at 50°C was also evaluated. Capability of the isolates to utilize various carbon sources was performed in ISP-2 agar medium with phenol red as indicator [20]. Carbon sources viz., fructose, lactose, starch, dextrose, rhamnose, mannitol, maltose, adonitol, arabinose and raffinose were used in this study. Identification of the isolates was made with reference to Bergey’s manual of Systematic Bacteriology [21] and Waksman [22].

### Screening of marine actinobacteria for antibacterial potential

Isolates collected from Minnie Bay were screened for antibacterial activity by cross streak method [23]. The isolates were cross streaked on SCA medium and incubated at room temperature for 5 days. After observing a good ribbon like growth of actinobacterial cultures, overnight cultures of *Proteus mirabilis* MTCC1429, *Escherichia coli* MTCC443, *Vibrio cholerae* MTCC3904, *Klebsiella pneumoniae* MTCC109, *Streptococcus pneumoniae* MTCC1935, *Enterococcus faecalis* MTCC439, *Pseudomonas aeruginosa* MTCC424, *Bacillus subtilis* MTCC441, *Staphylococcus aureus* MTCC96, *Shigella flexineri* MTCC1457, *Micrococcus luteus* MTCC1541 and *Salmonella typhi* MTCC734 were streaked at the right angle of actionobacterial cultures. Plates were again incubated at 28°C for 48 hrs and the zone of inhibition was documented. SCA plates without actinobacteria, but with simultaneous streaking of test organisms were maintained as control.

### Extraction of antibacterial compounds

Selected antagonistic actinobacterial isolates (*Streptomyces* sp. NIOT-VKKMA02, *Streptomyces* sp. NIOT-VKKMA26 and *Saccharopolyspora* sp. NIOT-VKKMA22) were inoculated into starch casein broth, and incubated on a shaker at 28°C for 7 days. After incubation, culture broths were filtered through Whatman No.1 filter paper to separate cell mass from the medium. The cell filtrate was mixed separately in ethyl acetate, ethyl alcohol, methanol and concentrated under pressure in a Buchi Rotavapor R-205 (Buchi Labortechnik AG, Switzerland) at 30°C. Further, the crude solvent extracts were screened for antibacterial activity against 12 clinical pathogens by well diffusion assay. A known quantity of 50 μg/well was loaded in Muller Hinton agar plates seeded with test organisms. Negative controls with solvents were also maintained. After overnight incubation at 37°C, the zone of inhibition was documented in millimeter. To authenticate the antibacterial property of crude extracts, screening assay was carried out in triplicates.

### Screening of marine actinobacteria for surfactant production

#### Hemolytic activity

Screening of isolates for hemolytic activity were performed in blood agar medium containing 5% (w/v) peptone, 3% (w/v) yeast extract, 5% (w/v) NaCl and 5% (v/v) human blood [24]. Plates were examined for hemolysis after incubation at 37°C for 5 days. Presence of clear zone around colonies signifies the potential of isolates for surfactant production.

#### Screening for lipase production

Aptitude of the isolates to synthesize extracellular lipase was monitored using ISP 2 medium with 1% (w/v) tributyrin with pH 7.4. A loopful of inoculum was streaked on to test agar plates and incubated at 30°C for 7 days. After incubation, the plates were examined for potential lipase producers by recording clear zone around colonies.

#### Production medium

Potential isolates (*Streptomyces* sp. NIOT-VKKMA02, *Streptomyces* sp. NIOT-VKKMA26 and *Saccharopolyspora* sp. NIOT-VKKMA22) for surfactant biosynthesis was further cultivated in production medium with 5% (w/v) peptone, 1% (w/v) yeast extract, 10% (w/v) glucose, 1% (w/v) NaCl, 0.5% (w/v) K_2_HPO_4_, 0.1% (w/v) FeSO_4_, 0.2% (w/v) Na_2_CO_3_ and 0.1% (w/v) MgSO_4_, with pH 7 and incubated at 28°C for 7 days on a shaker incubator at 200 rpm.

#### Drop collapsing test

Quantitative drop-collapse test to confirm surfactant production by potential isolates was performed as described by Youssef et al. [25]. Briefly, 0.02% (v/v) mineral oil was stacked on to 96 well microtitre plates and equilibrated for 1 h at 37°C. Subsequently, 5 μl of culture supernatant was added to the surface of oil and the shape of supernatant on oil surface was observed after 1 min. Culture supernatant that makes oil to collapse was documented as positive and supernatant remains beaded were scored as negative, which are scrutinized with distilled water as control.

#### Oil displacement test

Oil displacement assay was performed based on the methodology of Morikawa et al. [26]. Weathered crude oil 0.015% (v/v) was laid on 40 μl of Milli Q water in a sterile Petri plate. Subsequently, 10 μl of culture supernatant was gently added on the surface of oil film. Diameter and area of clear halo visualized under visible light were measured after 1 min.

### Emulsification assay

Emulsification activity was determined by the methodology reported by Paraszkiewicz et al. [27]. Kerosene and cell free supernatant was mixed in the final concentration of 1:1, vortexed vigorously for 2 min and incubated at room temperature for 24 h. Height of the emulsified layer and emulsification index was estimated as *E*_24_ = *H*_EL_*/H*_S_ × 100, where E_24_ is the emulsification activity after 24 h, *H*_EL_ the height of emulsified layer, and *H*_S_ is the height of total liquid column. The assay was performed in triplicate and compared with distilled water as control.

### Screening of marine actinobacteria for extracellular enzymes

#### Primary enzymatic screening

Screening of isolates were performed to determine its capability to yield industrially important enzymes such as lipase, amylase, protease, gelatinase, cellulase, DNase, urease and phosphatase with the methods adopted previously by Leon et al. [28]. Isolates were streaked on test agar medium with respective substrates such as starch, carboxymethyl cellulose (CMC), gelatin, tributyrin, casein, 40% urea, 0.2% DNA and phenolphthalein phosphate agar plates separately and incubated at room temperature for 5 days. After incubation, plates were flooded with respective indicator solution and the development of clear zone around the growth of organism was documented as positive results for enzyme activity.

### Secondary enzymatic screening

#### Amylase activity

Studies on amylase production with the potential isolates (*Streptomyces* sp. NIOT-VKKMA02, *Streptomyces* sp. NIOT-VKKMA26 and *Saccharopolyspora* sp. NIOT-VKKMA22) were performed by shake flask method. The production medium consisted of 1% (w/v) soluble starch, 0.2% (w/v) yeast extract, 0.5% (w/v) peptone, 0.05% (w/v) MgSO_4_, 0.05% (w/v) KH_2_PO_4_, 0.15% NaCl and 0.05% CaCl_2_ with pH 7. Isolates were inoculated into production medium and incubated in shaker incubator at 28°C for 7 days. After incubation, culture broth was filtered through Whatman No.1 filter paper and cell free supernatant was obtained by centrifugation at 10,000 rpm for 10 min. Amylase activity was determined by the amount of glucose equivalents released in medium. Briefly, 10 ml reaction mixture consisting of 0.5 ml cell free supernatant (CFS), 0.5 ml of 1% soluble starch dissolved in 0.1 M phosphate buffer (pH 7), remaining sterilized distilled water and incubated at 37°C for 15 min [29]. Reaction was stopped by adding 3, 5-dinitrosalicylic acid [30], and by boiling for 10 min. Concentration of released glucose was measured at 620 nm and the quantity was determined with glucose standard curve. One unit (U) of amylase activity was defined as the μg quantity of glucose equivalents liberated per min per ml of enzyme under controlled conditions.

### *Cellulase activity*

Cellulase activity was performed by shake flask method, with the medium composition of 0.5% (w/v) CMC, 0.2% (w/v) yeast extract, 0.5% (w/v) peptone, 0.05% (w/v) MgSO_4_, 0.05% (w/v) KH_2_PO_4_, 0.15% NaCl and 0.05% CaCl_2_ with pH 7. Prospective actinobacterial isolates (*Streptomyces* sp. NIOT-VKKMA02, *Streptomyces* sp. NIOT-VKKMA26 and *Saccharopolyspora* sp. NIOT-VKKMA22) were inoculated into production medium and incubated in shaker incubator at 28°C for 7 days. After incubation, culture broth was filtered through Whatman No.1 filter paper and cell free supernatant was obtained by centrifugation at 10,000 rpm for 10 min. Cellulase activity was determined by the amount of glucose equivalents released in medium. 10 ml reaction mixture consisting of 0.5 ml CFS, 0.5 ml of 0.5% CMC dissolved in 0.1 M phosphate buffer (pH 7), remaining sterilized distilled water and incubated at 37°C for 15 min [29]. Reaction was stopped by adding 3, 5-dinitrosalicylic acid [30], and by boiling for 10 min. Concentration of released glucose was measured at 620 nm and the quantity was determined with glucose standard curve. One unit (U) of cellulase activity was defined as μg quantity of glucose equivalents liberated per min per ml of enzyme under prescribed conditions.

### *Protease activity*

Potential of the isolates to synthesize protease was performed by shake flask method, with medium composition of 0.2% (w/v) soluble starch, 0.05% (w/v) peptone, 0.05% (w/v) glucose, 0.05% (w/v) yeast extract, 0.05% (w/v) casein, 0.02% (w/v) soyabean meal, 0.06% (w/v) (NH4)_2_SO_4_, 0.08% (w/v) CaCO_3_ and 0.05% NaCl with pH 7. Prospective actinobacterial isolates (*Streptomyces* sp. NIOT-VKKMA02, *Streptomyces* sp. NIOT-VKKMA26 and *Saccharopolyspora* sp. NIOT-VKKMA22) were inoculated into production medium and incubated in shaker incubator at 28°C for 7 days. After incubation, culture broth was filtered through Whatman No.1 filter paper and cell free supernatant was obtained by centrifugation at 10,000 rpm for 10 min. Protease activity was determined by incubating the reaction mixture containing 0.1 ml CFS and 0.9 ml of 2% casein in 0.1 M NaOH-KH_2_PO_4_ buffer (pH 7) at 37°C for 30 min. Reaction was stopped by addition of 1.5 ml of 1 M trichloroacetic acid. After 15 min, the mixture was centrifuged at 10,000 rpm for 10 min and the protein concentration in supernatant was determined according to the method of Lowry et al. [31]. One unit (U) of protease activity is equivalent to μg of tyrosine liberated per ml of enzyme under prescribed conditions.

### Molecular identification of potential strains

#### DNA isolation

Genomic DNA of *Streptomyces* sp. NIOT-VKKMA02, *Streptomyces* sp. NIOT-VKKMA26 and *Saccharopolyspora* sp. NIOT-VKKMA22 was isolated by following the modified procedure of Kutchma et al. [32]. Briefly, 2 ml of 72 hrs culture broth was centrifuged at 8,000 rpm for 5 minutes at room temperature and the pellets were washed with 1 ml TE buffer, suspended in TE buffer containing lysozyme with the final concentration of 4 mg/ml. The mixture was incubated at 37°C water bath for 3 hrs. Subsequently, 75 μl of 10% SDS and 125 μl of 5 M NaCl were added to cell pellet and incubated at 37°C for 30 min. Reaction tubes were later incubated at −40°C for 5 min and subsequently to 65°C water bath for 3 min. This step was repeated 3 times and the supernatant was collected by centrifugation at 8,000 rpm for 10 min at room temperature. To the supernatant, 50 μg/ml Proteinase K and 200 μg/ml RNase were added and incubated at 37°C for 30 min. Equal volume of phenol: chloroform: isoamyl alcohol (25:24:1) was added to the solution and mixed by inversion. After centrifugation at 8,000 rpm for 5 min, upper aqueous phase containing DNA was recovered and precipitated with two volumes of 95% ethanol by centrifugation at 13,000 rpm for 15 min. DNA pellet was dissolved in 50 μl of TE buffer and stored at −40°C for further use.

#### PCR amplification of 16S rRNA

Amplification of 16S rRNA was performed using universal primers 16Sf (5′ AGAGTTTGATCCTGGCTCAG 3′) and 16Sr (5′ GGTTACCTTGTTACGACTT 3′). Final volume of reaction was 25 μl, which comprised *Taq* buffer (1×), dNTP’s (200 μM) (MBI Fermentas, USA), forward and reverse primer (0.5 μM), MgCl_2_ (1.0 mM), *Taq* DNA polymerase (1.25 U; MBI Fermentas), template (1 μl) and remaining autoclaved Milli Q water. PCR was performed with the initial denaturation at 98°C for 3 min, followed by 30 cycles of reaction with denaturation at 94°C for 1 min; annealing at 53°C for 1 min; extension at 72°C and final extension at 72°C for 10 min. PCR amplified products were analyzed on 1.5% agarose gel along with DNA molecular weight marker (MBI Fermentas). Positive amplicons as judged by size were purified using QIAquick PCR purification kit (Qiagen, Germany) and sequenced on an ABI PRISM 377 genetic analyzer (Applied Biosystems, USA).

#### Phylogenic analysis

16S rRNA sequences of the potential strains (*Streptomyces* sp. NIOT-VKKMA02, *Streptomyces* sp. NIOT-VKKMA26 and *Saccharopolyspora* sp. NIOT-VKKMA22) was aligned manually in GenBank database with BLAST [33] and the sequences with 100-98% homology were considered for molecular taxonomy analysis. Multiple alignment of 16S rRNA sequences in this study and sequences in GenBank database was performed with CLUSTAL X program [34]. Phylogenetic trees were constructed by neighbor-joining and maximum-parsimony tree making methods in Molecular Evolutionary Genetic Analysis (MEGA version 5.0) [35] and bootstrap values based on 1,000 replication [36].

## Results

### Physico-chemical parameters

The details of sampling site and various physico-chemical properties of water samples collected from the site are provided in Table 1. In sampling site, DO value was observed to be 6.24 mg/l in both surface and bottom waters. Moreover, total nitrogen level was also found to a significant level (12.4 and 15.2 μmol/l) in surface and bottom waters, respectively. Sampling location was sloppy, muddy and was noticed with a wide diversity of marine life including flora, fauna and microbes.

**Table 1:** Physico-chemical parameters of study area (Minnie Bay)

**Parameters**	**Description**	**Description**	**Units**
Study area	Minnie Bay	Minnie Bay	
Latitude (N)	11° 38’ 42.8” N	11° 38’ 42.8” N	DD MM SS
Longitude (E)	92° 42’ 30.7” E	92° 42’ 30.7” E	DD MM SS
Year	2011	2011	YYYY
Month	May	May	Mon
Zone	Near shore	Near shore	
Source	Surface	Bottom	
Tide	Low Tide	Low Tide	
Atmospheric temperature	31.10		°C
**Water Quality**			
Water temperature	31.0	30.4	°C
pH	8.16	8.14	
Salinity	31.64	31.73	PSU
CO_3_^2-^	15.60	10.8	(mg/l)
HCO_3_^-^	21.96	35.38	(mg/l)
Dissolved Oxygen	6.24	6.24	(mg/l)
Biochemical Oxygen Demand	2.90	2.81	(mg/l)
Suspended solid concentration	40.56	75.65	(mg/l)
Nitrite	0.04	0.16	(μmol/l)
Nitrate	0.75	0.72	(μmol/l)
Ammonia	0.12	0.42	(μmol/l)
Total Nitrogen	12.4	15.2	(μmol/l)
Inorganic Phosphate	0.18	0.18	(μmol/l)
Total Phosphorous	0.56	0.65	(μmol/l)
Silicate	4.89	4.55	(μmol/l)

### Characterization of isolates

**Figure 2:**
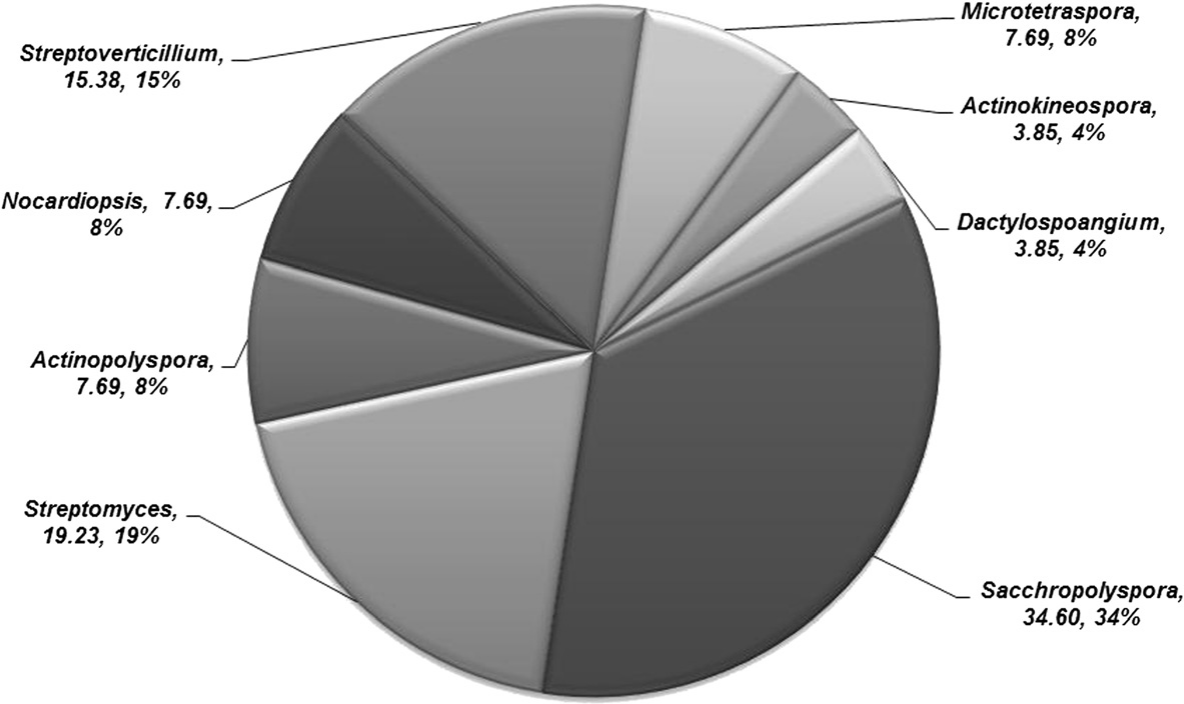
Percentage frequency of isolated actinobacteria genera.

**Figure 3:**
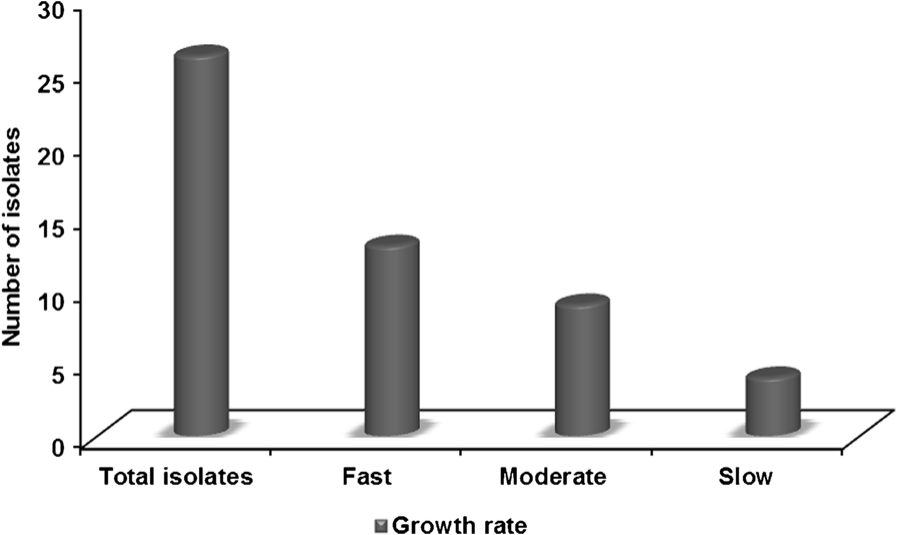
Growth rate of actinobacteria isolated from A & N Islands.

Sediment samples were collected during low tide and a total of 26 actinobacteria were isolated using SCA medium with nalidixic acid prepared in aged seawater. All isolates were identified at generic level based on the colony, microscopic observations and biochemical characteristics. Morphological and cultural characteristics revealed that, maximum of (65.39%) isolates fit in to greenish, blue and grey colour series. Of 26 isolates, 34.60% (n = 9) isolates were allocated to the genus *Saccharopolyspora*, 19.23% (n = 5) isolates were assigned as genus *Streptomyces* and remaining isolates as *Streptoverticillium* (n = 4), *Actinopolyspora* (n = 2), *Nocardiopsis* (n = 2), *Microtetraspora* (n = 2), *Actinokineospora* (n = 1) and *Dactylosprangium* (n = 1). Percentage frequency of isolates is shown in (Figure 2). Present study revealed that; of the total isolates, *Saccharopolyspora* and *Streptomyces* were found to be the dominant genera belongs to the class Actinobacteria and order Actinomycetales. In this study, majority of the isolates determined aerial coiled mycelia and spores arranged in chains. Among 26 isolates, 8 genera were identified and each genus was distinguished by their spore, mycelia and aerial hyphae. Isolates were screened for their optimum growth on SCA medium, of 26 isolates; 13 isolates (50%) revealed fast growth, 9 isolates (34.6%) exhibited moderate growth and minimum of 4 isolates (15%) were determined as slow growers (Figure 3). Morphological, physiological, biochemical, cultural characteristics and utilization of carbon sources of the isolates are given in Tables 2 and 3. Of 26 actinobacterial isolates, 12 isolates produced melanin, 23 isolates displayed distinctive reverse side pigment and 6 isolates produced diffusible pigments.

**Table 2:** Phenotypic characteristics of selected actinobacteria from A & N Islands

**Properties**	***Streptomyces *sp. NIOT-VKKMA246**	***Streptomyces *sp. NIOT-VKKMA326**	***Saccharopolyspora *sp. NIOT-VKKMA1713,4522**	***Streptoverticillium *sp. NIOT-VKKMA16,234**
**Morphological characteristics**				
Spore morphology	Chain	Spiral	Hook	Chain
Colour of aerial mycelium	Green	Dark grey	Blue	Greenish grey
Colour of substrate mycelium	Grey	Brown	Brown	Grey
Soluble pigment	Greenish brown	Brown	-	-
Spore mass	Green	Dark grey	Blue	Green
**Biochemical characteristics**				
Gram staining	+	+	+	+
Indole production	-	-	-	-
Methyl Red	+	-	-	+
Voges Proskauer	-	-	-	-
Citrate utilization	+	+	+	+
H_2_S production	-	+	+	-
Nitrate reduction	+	+	+	+
Urease	+	+	+	+
Catalase	-	+	+	-
Oxidase	+	-	-	+
Melanin production	-	+	+	-
Starch hydrolysis	+	+	+	-
Haemolysis	+	+	+	+
Triple sugar iron	alk/alk	alk/alk	alk/alk	alk/alk
Survival at 50°C	Moderate	Good	Good	Moderate
**Carbon source utilization**				
Starch	+	+	+	-
Dextrose	-	+	+	-
Fructose	+	+	+	+
Maltose	+	+	+	+
Mannitol	+	+	+	+
**pH**				
5	+	-	-	+
6	+	+	+	+
7	+	+	+	+
8	+	+	+	+
9	+	+	+	+
10	+	+	+	+
11	+	+	+	+
**NaCl tolerence (%)**				
5	+	+	+	+
10	+	+	+	+
15	+	+	+	+
20	+	+	+	+
25	+	+	+	+
30	+	-	-	+

**Table 3:** Phenotypic characteristics of selected actinobacteria from A & N Islands

**Properties**	***Actinopolyspora *NIOT-VKKMA818**	***Nocardiopsis *NIOT-VKKMA525**	***Microtetraspora *NIOT-VKKMA1719**	***Dactylospoangium *NIOT-VKKMA21**
**Morphological characteristics**				
Spore morphology	Long elongated	Coccoid	Short	Finger shaped
Colour of aerial mycelium	Pale yellow	Dull brown	Creamy white	Greenish black
Colour of substrate mycelium	Brown	Brown	Brown	-
Soluble pigment	Greenish brown	Brown	-	-
Spore mass	Pale yellow	Dull brown	Creamy white	Greenish black
**Biochemical characteristics**				
Gram staining	+	+	+	+
Indole production	-	-	+	-
Methyl Red	+	+	-	-
Voges Proskauer	+	-	-	-
Citrate utilization	+	-	+	-
H_2_S production	+	+	-	+
Nitrate reduction	+	-	-	+
Urease	-	+	+	+
Catalase	+	+	+	+
Oxidase	+	+	+	+
Melanin production	+	+	+	-
Starch hydrolysis	+	+	+	-
Haemolysis	+	+	+	-
Triple sugar iron	-	alk/alk	alk/alk	-
Survival at 50°C	Excellent	Excellent	-	-
**Carbon source utilization**				
Starch	+	+	+	-
Dextrose	+	+	+	+
Fructose	-	+	+	-
Maltose	+	+	-	+
Mannitol	-	+	-	+
**pH**				
5	-	-	-	+
6	+	+	+	+
7	+	+	+	+
8	+	+	+	+
9	+	+	+	+
10	+	+	+	+
11	+	+	+	+
**NaCl tolerence (%)**				
5	+	+	+	+
10	+	+	+	+
15	+	+	+	+
20	+	+	+	+
25	-	+	-	+
30	-	+	-	+

### Antibacterial potential of isolates

**Figure 4:**
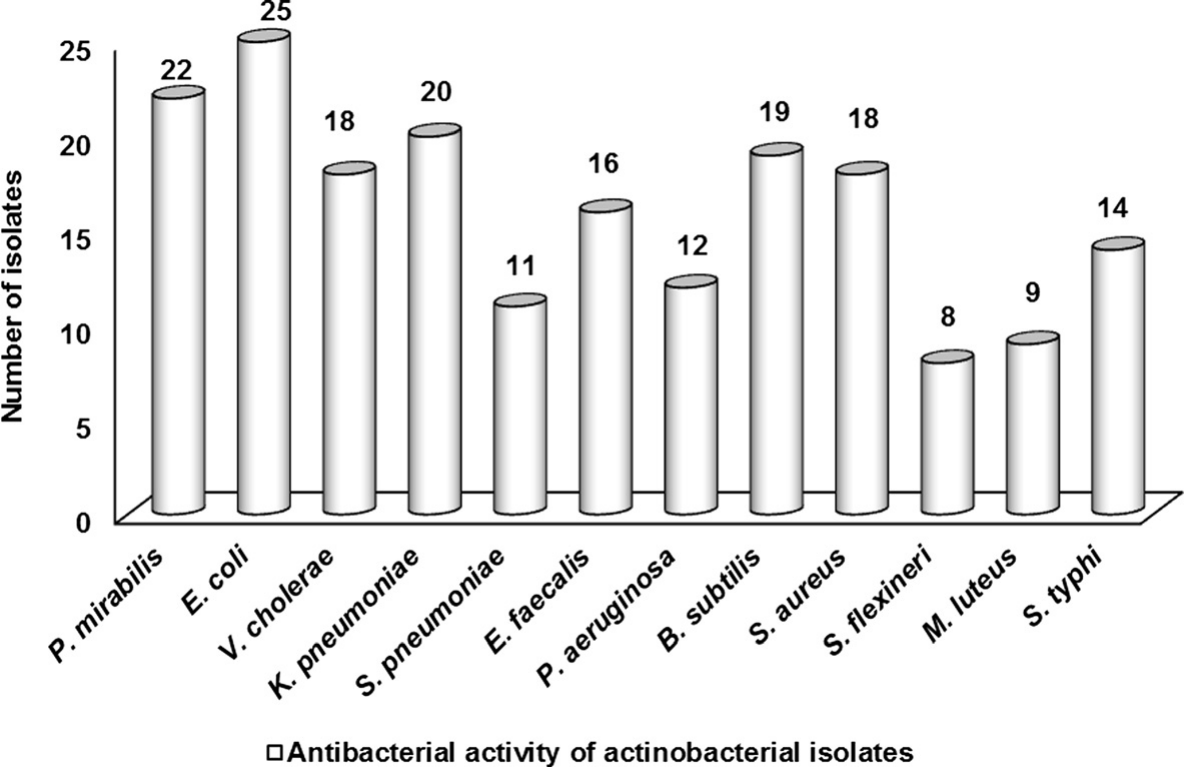
Antibacterial activity of actinobacterial isolates from A & N Islands.

Isolates were analyzed against 12 clinical pathogens and the extent of antibacterial activity was varied among the actinobacterial isolates (Figure 4). Of 26 isolates, 96% exhibited appreciable inhibitory activity against Gram negative bacteria and 73% acted against Gram positive bacteria. Remaining 23% revealed excellent antibacterial activity against both Gram positive and Gram negative bacteria. However, strain *Streptomyces* sp. NIOT-VKKMA02 was found to have broad spectral antibacterial activity and was further investigated by 3 different solvent extracts. Of which, ethyl acetate extract disclosed maximum inhibitory activity against all pathogens tested than methanol and ethanol extracts. Antibacterial efficacy of ethyl acetate extract from *Streptomyces* sp. NIOT-VKKMA02 against clinical pathogens is depicted in Table 4.

**Table 4:** Antimicrobial activity of potential isolates with different solvents

**Test organisms**	***Streptomyces *sp. NIOT-VKKMA02**			***Streptomyces *sp. NIOT-VKKMA26**			***Saccharopolyspora *sp. NIOT-VKKMA22**		
	**Zone of inhibition (mm)**								
	**Ethyl acetate**	**Methanol**	**Ethanol**	**Ethyl acetate**	**Methanol**	**Ethanol**	**Ethyl acetate**	**Methanol**	**Ethanol**
*P. mirabilis*	21	19	13	17	13	8	16	9	8
*E. coli*	26	23	11	22	17	14	24	7	-
*V. cholerae*	20	17	12	18	11	11	15	12	10
*K. pneumoniae*	17	14	14	16	9	7	13	10	-
*S. pneumoniae*	37	34	16	26	26	21	22	19	15
*E. faecalis*	33	28	14	20	12	11	15	-	-
*P. aeruginosa*	14	10	11	-	9	-	7	-	-
*B. subtilis*	42	36	19	33	-	-	21	14	7
*S. aureus*	48	39	21	24	-	-	19	-	-
*S. flexineri*	12	10	-	18	8	-	13	7	-
*M. luteus*	11	9	-	-	-	-	-	-	-
*S. typhi*	34	26	14	19	-	-	-	-	-

### Potential of isolates in surfactant production

Actinobacterial isolates were studied for their ability to synthesize surface active molecules. Isolates were processed with series of tests viz., streaking in blood agar, lipolytic activity, drop collapsing test, oil displacement assay and emulsification activity. Of 26 isolates, maximum of 20 (77%) revealed positive results for hemolycin production by forming clear zone around the colonies in blood agar medium. In lipolytic assay, clear zone was observed around the colonies on tributyrin agar plates by lipase enzyme production. Isolates *Streptomyces* sp. NIOT-VKKMA02, *Streptomyces* sp. NIOT-VKKMA26 and *Saccharopolyspora* sp. NIOT-VKKMA22 illustrated the maximum comprehensible zones with 25 mm, 17 mm and 13 mm, respectively. Moreover, the same proportion of isolates determined positive results for drop collapsing and oil displacement assays by forming flat drop and increasing the surface area, respectively. These results confirmed the capability of isolates to synthesize surface active molecules of environmental importance. Actinobacterial strain *Streptomyces* sp. NIOT-VKKMA02 revealed best result for oil replacement area with 36.29 cm^2^. Emulsification activity (*E*_24_) of the surfactant from *Streptomyces* sp. NIOT-VKKMA02 was measured with kerosene and CFS, *E*_24_ ranged from 1.8-63.6%. Emulsification activity of the potential isolate was perceived from first day of incubation and demonstrated highest emulsification activity on 7^th^ day.

### Growth characteristics of the isolates

Isolates were screened for their growth at various pH and NaCl levels. Unexpectedly, all isolates exhibited excellent growth in the pH range of 6–11 and 69.23% isolates displayed good growth at acidic pH (pH-5). However, of 26 isolates, 61.5% isolates recorded good growth in 25% NaCl and 18% displayed excellent growth in 30% NaCl. With reference to the growth studies, it was established that, all isolates in this study were halophilic and alkalitolerant.

### Screening of extracellular enzymes

**Figure 5:**
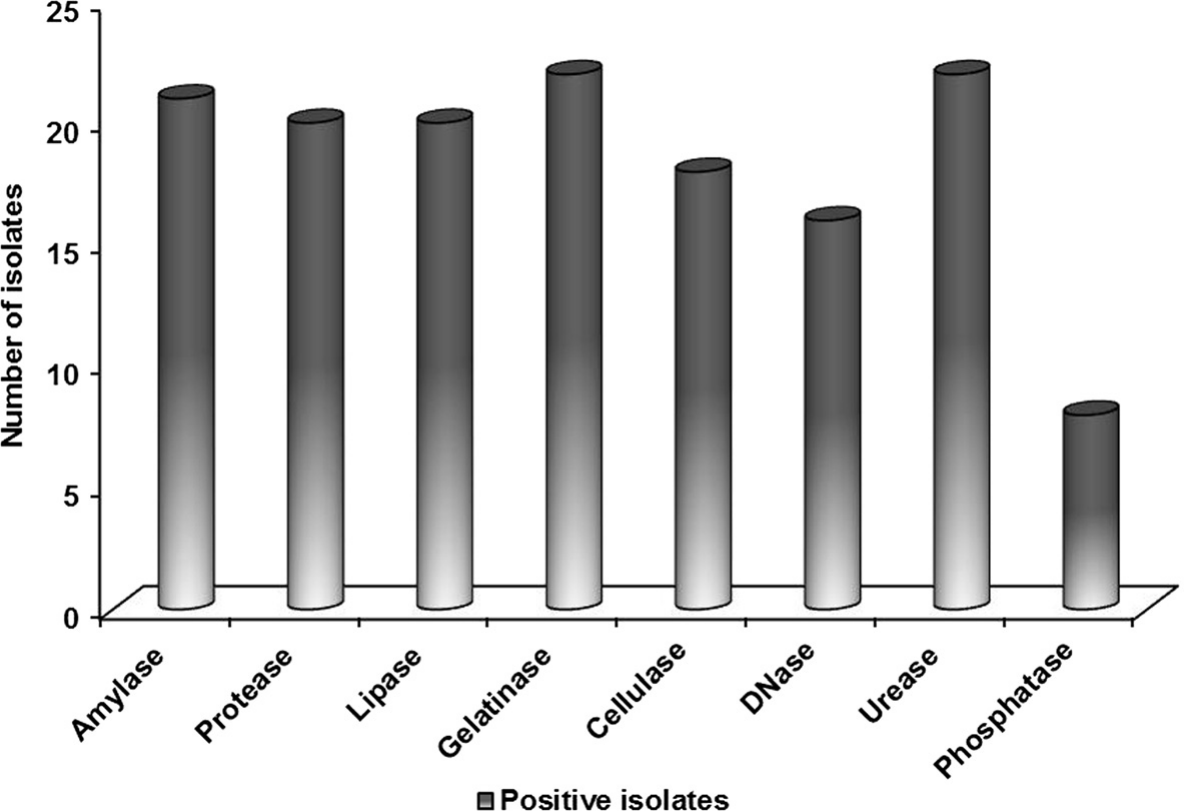
Multi-enzyme activity of actinobacterial isolates from A & N Islands.

No studies on characterization of extracellular enzyme production from marine actinobacteria of A & N Islands have been reported. Of 26 isolates, 22 isolates were found to synthesize gelatinase and urease, 21 isolates demonstrated amylolytic activity, 20 isolates exhibited proteolytic and lipolytic activity and 18 isolates displayed cellulolytic activity. Interestingly, 16 isolates exhibited excellent DNase activity and 8 isolates revealed positive for alkaline phosphatase (Figure 5). To our recognition, 13 isolates exhibited constructive results in the production of 8 extracellular enzymes of industrial importance. *Streptomyces* sp. NIOT-VKKMA02, *Streptomyces* sp. NIOT-VKKMA26 and *Saccharopolyspora* sp. NIOT-VKKMA22 exhibited elevated enzymatic activity for all 8 industrial enzymes. Consequently, these potent isolates were subjected for the detailed characterization on industrially potent enzymes like amylase, cellulase and protease. Production of enzymes by the potent isolates was achieved by submerged fermentation and their enzymatic activities are shown in Table 5. As specified in the table, isolate *Streptomyces* sp. NIOT-VKKMA02 proved maximum amylolytic activity (R/r = 4.3), proteolytic activity (R/r = 3.1) and cellulolytic activity (R/r = 2.8). Spectrophotometric analysis on amylase production in *Streptomyces* sp. NIOT-VKKMA02, *Streptomyces* sp. NIOT-VKKMA26 and *Saccharopolyspora* sp. NIOT-VKKMA22 were found to be in higher side with 13.27 U/ml, 9.85 U/ml and 8.03 U/ml respectively. No studies have ever been reported with that of utmost production in industrially potent enzymes by our isolates. Moreover, production of cellulase by *Streptomyces* sp. NIOT-VKKMA02, *Streptomyces* sp. NIOT-VKKMA26 and *Saccharopolyspora* sp. NIOT-VKKMA22 were also found to be in elevated phase with 7.75 U/ml, 5.01 U/ml and 2.08 U/ml, respectively. Quantitative assay of proteolytic activity revealed that *Streptomyces* sp. NIOT-VKKMA02, *Streptomyces* sp. NIOT-VKKMA26 and *Saccharopolyspora* sp. NIOT-VKKMA22 produced 11.34 U/ml, 6.89 U/ml and 3.51 U/ml of protease enzyme, respectively.

**Table 5:** Enzyme activity of potential isolates

**Isolates**	Amylolytic zone (R/r)*	**Amylase (IU/ml)**	**Cellulolytic zone (R/r)**	**Cellulase (IU/ml)**	**Proteolytic zone (R/r)**	**Protease (IU/ml)**
*Streptomyces* sp. NIOT-VKKMA02	4.3	13.27	2.8	7.75	3.1	11.34
*Streptomyces* sp. NIOT-VKKMA26	3.6	9.85	2.1	5.01	2.3	6.89
*Saccharopolyspora* sp. NIOT-VKKMA22	3.1	8.03	1.7	2.08	1.9	3.51

*R: Hydrolyzed zone diameter; r: Growth zone diameter.

### Molecular identification and phylogenies of potential isolates

**Figure 6:**
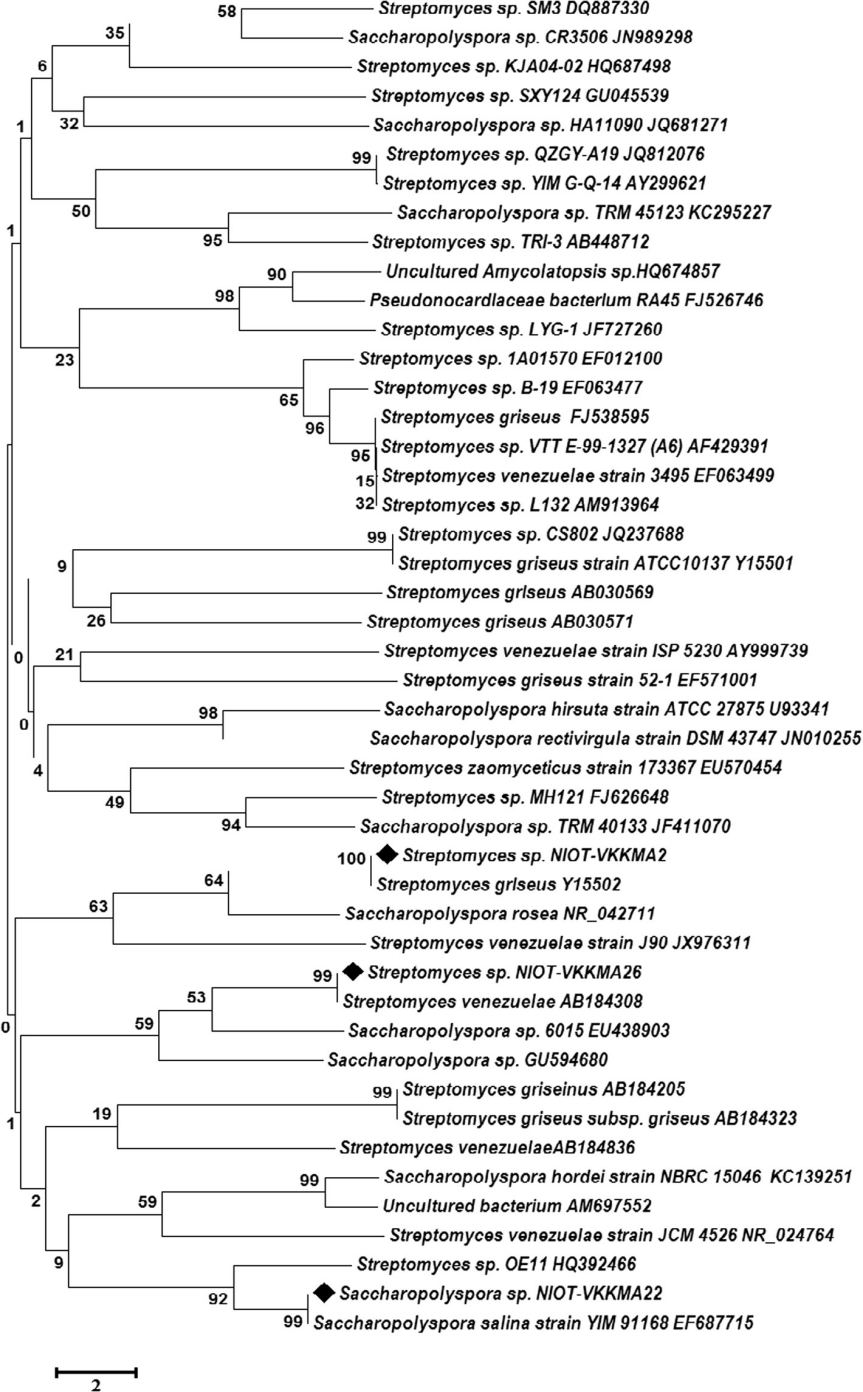
**Phylogenetic tree based on 16S rRNA sequences using neighbor-joining method for the strains NIOT-VKKMA02, NIOT-VKKMA26 and NIOT-VKKMA22.** Branch distances represent nucleotide substitution rate and scale bar represents the number of changes per nucleotide position.

Phylogenetic relationships of our isolates were ascertained based on the 16S rRNA sequence similarity with reported strains using BLAST sequence similarity search. Upon analysis, it was established that the deduced 16S rRNA sequences of *Streptomyces* sp. NIOT-VKKMA02 [GenBank: KC593858] was highly homologous (100%) with reported sequences of *Streptomyces griseus* [GenBank: Y15502]. Sequence analysis also specified that 16S rRNA sequences of *Streptomyces* sp. NIOT-VKKMA02 was closely related to the phylogenetic neighbors; *Streptomyces flaveus*, *Streptomyces flavolimosus* and *Streptomyces flavogriseus* with sequence similarity of 100 and 99%, respectively. Phylogenetic analysis based on neighbor-joining tree (Figure 6) further revealed that strain NIOT-VKKMA02 formed a distinct branch with *Streptomyces griseus*. 16S rRNA sequences of *Streptomyces* sp. NIOT-VKKMA26 [GenBank: KC593859] was highly homologous (100%) with reported sequences of *Streptomyces venezuelae* [GenBank: AB184308]. Sequence analysis also indicated that 16S rRNA sequence of *Streptomyces* sp. NIOT-VKKMA26 was highly homologous to the phylogenetic neighbors; *Streptomyces phaeochromogenes*, *Streptomyces zaomyceticus*, *Streptomyces exfoliatus* and *Streptomyces tateritius* with sequence similarity of 100 and 99%. Neighbor-joining tree also disclosed that strain NIOT-VKKMA26 forms a single cluster with *Streptomyces venezuelae* (Figure 6). The sequences of *Saccharopolyspora* sp. NIOT-VKKMA22 [GenBank: KC593860] also established 100% homology with the previous report of *Saccharopolyspora salina* [GenBank: EF687715]. BLAST analysis also indicated that 16S rRNA sequences of *Saccharopolyspora* sp. NIOT-VKKMA22 was found extremely related to the phylogenetic neighbors; *Saccharopolyspora rosea*, *Saccharopolyspora halophila*, *Saccharopolyspora pogona* and *Saccharopolyspora erythraea* with the similarity between 95 and 94%. Neighbor-joining tree (Figure 6) also disclosed a distinct cluster between NIOT-VKKMA22 and *Saccharopolyspora salina.* Actinobacterial species switched to different clusters indicates the divergence among organisms and degree of divergence in sequences. 16S rRNA sequence analysis clearly concluded that our isolates *Streptomyces* sp. NIOT-VKKMA02, *Streptomyces* sp. NIOT-VKKMA26 and *Saccharopolyspora* sp. NIOT-VKKMA22 are as *Streptomyces griseus*, *Streptomyces venezuelae* and *Saccharopolyspora salina,* respectively. No report accomplished the presence/occurrence of these marine actinobacreia from this emerald Island and further studies on fatty acid profiling and GC content analysis among these strains will be the added authentication to confirm our isolates as novel.

### Description for *Streptomyces griseus* NIOT-VKKMA02

Gram positive, non-acid fast, non-motile, aerobic, very long rods and filamentous organism, spores on aerial mycelium, looped or spiral chains observed by cover-slip method and evaluated by phase contrast microscope. Competent to generate yellow pigment and exhibited optimum growth under aerobic conditions at pH 8.0 and NaCl tolerance was at 5-15% (w/v). Accordingly, it was considered as alkalitolerant and moderate halophilic. Illustrated differences in carbon utilization, able to utilize all sugars except salicilin and arabinose, positive results for methyl red test, nitrate reduction test, citrate utilization, urea hydrolysis, cytochrome oxidase, catalase test, gelatin hydrolysis and esculin. Exhibited broad antibacterial spectrum against investigated clinical pathogens.

### Description for *Streptomyces venezuelae* NIOT-VKKMA26

Gram positive, non-acid fast, non-motile, aerobic, very long rods and filamentous organism, spiral spore-forming hyphae, spores on aerial mycelium in straight and hooked mode as observed using cover-slip method and evaluated by phase contrast microscope. Soluble pigments were found deficient and exhibited optimum growth under aerobic conditions at pH 8.0 and optimum NaCl concentration at 5-20% (w/v). Therefore, it was considered as alkalitolerant and moderate halophilic. Showed divergence in carbon utilization, able to utilize sucrose, fructose, mannitol, maltose, lactose, rhamnose and raffinose, proved positive results for methyl red test, Voges-Proskuer, nitrate reduction test, citrate utilization, urea hydrolysis, cytochrome oxidase, catalase test, gelatin hydrolysis, lipid hydrolysis, hemolysis, starch hydrolysis and esculin hydrolysis. Exhibited broad antibacterial spectrum against examined clinical pathogens.

### Description for *Saccharopolyspora salina* NIOT-VKKMA22

Aerobic, non-acid fast, extensively branched substrate hyphae fragmented into rod-shaped, non-motile elements and aerial hyphae differentiated into bead-like chains of spores and carry long chains of spores in a spiral arrangement. Able to utilize variety of organic compounds; arabinose, adonitol, glucose, fructose, mannose, cellobiose, lactose, fucose, arabitol, maltose, sucrose, trehalose, inulin, raffinose, rhamnose, *N*-acetylglucosamine, aesculin, starch, glycogen and potassium gluconate. Proficient to degrade starch, cellulose, casein and gelatin. Good growth in the range of 5-15% (w/v) NaCl. Negative for oxidase and nitrate reduction, positive for catalase, alkaline phosphatase and urease.

## Discussion

Research on marine actinobacteria from A & N Islands is very scanty and till date these Island resources have not been properly explored to identify novel microorganisms with potential biological properties. With this outlook, the present research has been initiated to identify novel actinobacterial isolates from marine sediments of Minnie Bay, South Andaman Island. In this study, actinobacterial strains were isolated using modified growth medium. It has already been reported the usage of aged seawater enriched modified media for the isolation of marine actinobacteria [13]. Various selective media were used for isolation and enumeration of actionobacteria [16,37].

*Streptomyces* and *Saccharopolyspora* were found to be the dominant genera and their occurrence in Bay of Bengal was already reported [6]. Frequency and dominance of *Streptomyces* in various sources have also been reported [11,38,39]. Majority of the isolates in this study possessed coiled mycelia and the same morphology has been reported by Roes and Meyer [40]. Spore morphology is considered as one of the important characteristic features in actinobacterial identification and it varies among the genus and species [13,41]. Moreover, the results acquired in this study have been outlined in Bergey’s Manual of Systematic Bacteriology [21] and Laboratory manual for identification of actinomycetes [42]. Diversity of actinobacteria in Chesapeake Bay was also reported similar to our mode of observations [43]. Based on growth studies, it was made known that majority of the isolates grew well in modified SCA medium. This has been already reported in actinobacterial community isolated from Bay of Bengal [13]. Varied pigment production pattern was also observed among our isolates. Shirling and Gottileb [18] reported that the pigmentation prototype can be used as markers for identification. Moreover, cultural characteristics and utilization of carbon by the isolates in different media (ISP-2 to ISP-7) also play a major role in identification of actinobacteria to generic level. It is also proved that different physiological characteristics will certainly influence the growth rate of actinobacteria [44,45].

Actinobacteria are the main basis of clinically significant antibiotics [46]. Recent reports revealed that about 4,607 patents have been issued on actinobacteria related product and process. The genus *Saccharopolyspora* of *Pseudonocardiaceae* family is recognized for producing various antibiotics like vancomycin, erythromycin and rifamycins [47]. Majority of our isolates exhibited appreciable antibacterial activity against tested clinical pathogens. Of three solvents used, ethyl acetate extract of *Streptomyces* sp. NIOT-VKKMA02 determined better inhibitory activity. Earlier report [48] also revealed the effectiveness of ethyl acetate extracts from actinobacteria for antibacterial studies with that of other solvents. For the first of its kind, Grein and Meyers [49] have reported on antagonistic marine actinobacteria. Of their 66 isolates from marine sediments of New Jersey and Florida, 50% demonstrated antibiotic activity against Gram positive and Gram negative bacteria. Modest information on antimicrobial potential of marine actinobacteria from A & N Islands was previously reported. Of 88 marine actinobacterial isolates, only three isolates revealed noticeable antibacterial activity among test pathogens [11]. However, another report [12] disclosed that, of 42 isolates, only limited bioactivity (58.4%) was observed among test pathogens studied. As on date, our report will be the first to reveal a detailed study on antagonistic activity of marine actinobacteria from A & N Islands against both Gram positive and Gram negative eubacteria. In this study, majority of the isolates dominated in antibacterial potential against test pathogens. The reason may be the complex biochemical pathways adopted by our isolates due to the available nutrients and osmotic flux in sampling site.

Surfactants are amphiphilic compounds, produced by microorganisms of various classes including glycolipids, lipopeptides, fatty acids, phospholipids, neutral lipids and lipopolysaccharides [50]. Applications of surfactants includes excellent detergency, emulsification, foaming, wetting, penetrating, thickening, microbial growth enhancements, metal sequestering and oil recovering. Surfactants are promising compounds and offer several advantages over chemically synthesized surfactants due to its lower toxicity, biodegradability and ecological acceptability [51]. To our credit, *Streptomyces* sp. NIOT-VKKMA02 was found to have excellent emulsification property. Marine actinobacteria are good candidates for surfactant production, bioremediation and biodegradation [51]. Halotolerant *Streptomyces* was reported to be a good surfactant producer [52]. Based on literature survey, our study stands first in reporting surfactant production from marine actinobacteria of A & N Islands.

Growth survival studies of our isolates also accomplished to withstand in varied NaCl and pH levels. Based on previous reports, majority of the actinobacterial species isolated from marine sediments were moderate alkaliphilic and moderate halophilic in nature [6,10,11]. To cope with the external stress, these organisms have developed adaptive metabolic features to survive under extreme conditions [52]. *Nesterenkonia alba* sp. nov., an alkaliphilic actinobacterium was reported to grow optimally at pH 9–10 [53]. Chen et al. [54] also reported a halophilic marine actinomycete, *Nocardiopsis litoralis* sp. nov., isolated from a sea anemone.

Actinobacteria are physiologically diverse group in synthesizing various enzymes and metabolic products of industrial interest and are well recognized to produce most valuable pharmaceuticals and agrochemicals [55]. Marine actinobacteria isolated from East and West coast of India were reported in the production of various industrial enzymes [52]. Upon characterization for industrially potential enzymes, results from the potential isolates of our study revealed highly competent enzyme activity with that of previous reports. Bernfield [29] isolated several actinobacteria from marine sediments of the Central and West coast of Peru with multienzyme activity. Selvam et al. [56] reported 6.48 U/ml of amylase production from actinomycetes isolated from South Indian coastal region. While comparing with this result, *Streptomyces* sp. NIOT-VKKMA02 synthesized 13.27 U/ml of protease enzyme, which is two fold increases to that of previous report and the same augment was also recorded in cellulase production by the same strain. Hung-Der and Kuo-Shu [57] reported that the actinomycete, *Streptomyces* transformant T3-1 produced 2.6 U/ml of thermostable cellulase. Estimation of protease enzyme production also determined higher production level with the potential isolate. Ramesh et al. [10] 2009 reported that, *Streptomyces fungicidicus* MML1614 isolated from Bay of Bengal produced 7.5 U/ml of thermostable alkaline protease. These results on enzymatic production authenticated the capability of our isolate to over synthesize the valuable enzymes of industrial importance. Phylogenetic analyses also make known that *Streptomyces* sp. NIOT-VKKMA02, *Streptomyces* sp. NIOT-VKKMA26 and *Saccharopolyspora* sp. NIOT-VKKMA22 form a separate cluster with *Streptomyces griseus*, *Streptomyces venezuelae* and *Saccharopolyspora salina,* respectively. To the best of our knowledge, this is the first report on detailed characterization on enzymes with industrial and pharmaceutical importance from three novel marine actinobacteria of A & N Islands.

## Conclusions

In the current scenario, both academic and industrial research mainly focuses on marine microorganisms due to its impulsive potential. These credentials initiate the present research in search of salt and alkali tolerant novel actinobacteria from unexplored A & N Islands. Our study would be the first instance in comprehensive characterization of marine actinobacteria for industrial and pharmaceutical byproducts. Enhanced salt, pH and temperature tolerance of the isolates along with their capacity to secrete commercially valuable primary and secondary metabolites emerges an attractive feature of these organisms. Further, molecular characterization approach on these biological molecules will certainly bring out a new horizon in elevated production and can avoid complex downstream process associated with conventional methods. It is concluded that very frequent and systematic screening of marine actinobacteria from different sources and locations in A & N Islands may facilitate us to isolate and characterize more novel species with admirable bioactive compounds of interest.

## Competing interests

The authors declare that they have no competing interest.

## Authors’ contribution

Research concept and the experiments were performed by BM and LAR, NVV and RK analyzed the data and reviewed the manuscript. All authors approved the final manuscript.
